# Supplementary material for: Complex‐centric proteome profiling by SEC‐SWATH‐MS
Source: Mol Syst Biol. 2019 Jan 14;15(1):e8438. doi: 10.15252/msb.20188438 (PMC6346213; doi:10.15252/msb.20188438)
Supplement: Supplementary file 7 — Dataset EV6 [file MSB-15-e8438-s007.zip › feature_plots_bioplex/Q8IXJ6.pdf]

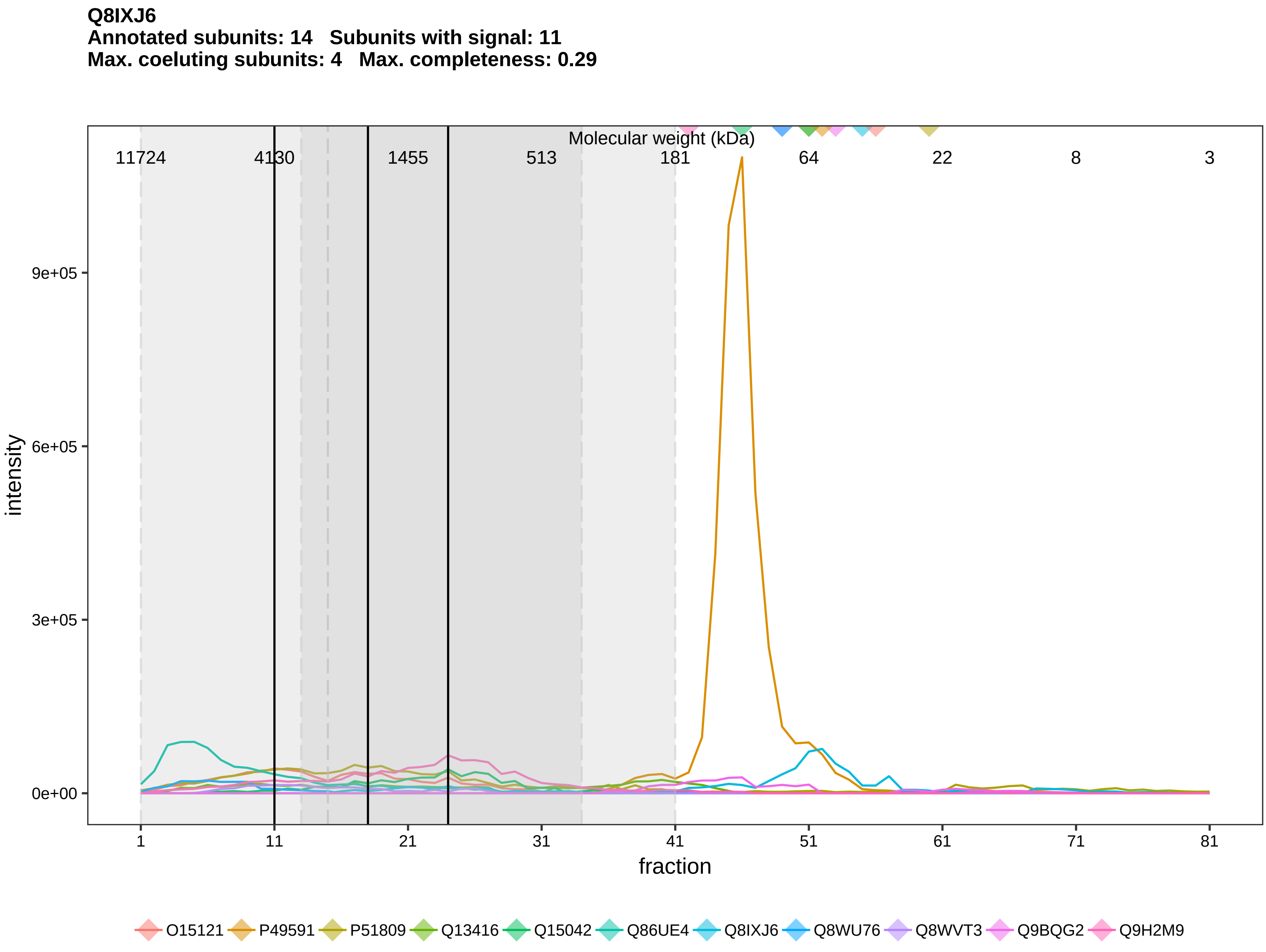
Q8IXJ6
Annotated subunits: 14 Subunits with signal: 11
Max. coeluting subunits: 4 Max. completeness: 0.29
Molecular weight (kDa)
11724
4130
1455
513
181
64
22
8
3
9e+05
6e+05
3e+05
0e+00
intensity
1
11
21
31
41
51
61
71
81
fraction
O15121
P49591
P51809
Q13416
Q15042
Q86UE4
Q8IXJ6
Q8WU76
Q8WVT3
Q9BQG2
Q9H2M9
